# Normothermic Regional Perfusion in Controlled Donation After Circulatory Death Liver Transplantation: A Systematic Review and Meta-Analysis

**DOI:** 10.3389/ti.2024.13263

**Published:** 2024-08-23

**Authors:** Carly Mastrovangelis, Charles Frost, Amy Hort, Jerome Laurence, Tony Pang, Henry Pleass

**Affiliations:** ^1^ Westmead Clinical School, Faculty of Medicine and Health, The University of Sydney, Sydney, NSW, Australia; ^2^ Department of Surgery, Westmead Hospital, Westmead, NSW, Australia; ^3^ Department of Surgery, Royal Prince Alfred Hospital, Sydney, NSW, Australia; ^4^ Surgical Innovations Unit, Westmead Hospital, Westmead, NSW, Australia

**Keywords:** liver transplantation, donation after circulatory death, normothermic regional perfusion, cDCD, NRP

## Abstract

Liver grafts from controlled donation after circulatory death (cDCD) donors have lower utilization rates due to inferior graft and patient survival rates, largely attributable to the increased incidence of ischemic cholangiopathy, when compared with grafts from brain dead donors (DBD). Normothermic regional perfusion (NRP) may improve the quality of cDCD livers to allow for expansion of the donor pool, helping to alleviate the shortage of transplantable grafts. A systematic review and metanalysis was conducted comparing NRP cDCD livers with both non-NRP cDCD livers and DBD livers. In comparison to non-NRP cDCD outcomes, NRP cDCD grafts had lower rates of ischemic cholangiopathy [RR = 0.23, 95% CI (0.11, 0.49), p = 0.0002], primary non-function [RR = 0.51, 95% CI (0.27, 0.97), p = 0.04], and recipient death [HR = 0.5, 95% CI (0.36, 0.69), p < 0.0001]. There was no difference in outcomes between NRP cDCD donation compared to DBD liver donation. In conclusion, NRP improved the quality of cDCD livers compared to their non-NRP counterparts. NRP cDCD livers had similar outcomes to DBD grafts. This provides further evidence supporting the continued use of NRP in cDCD liver transplantation and offers weight to proposals for its more widespread adoption.

## Graphical Abstract

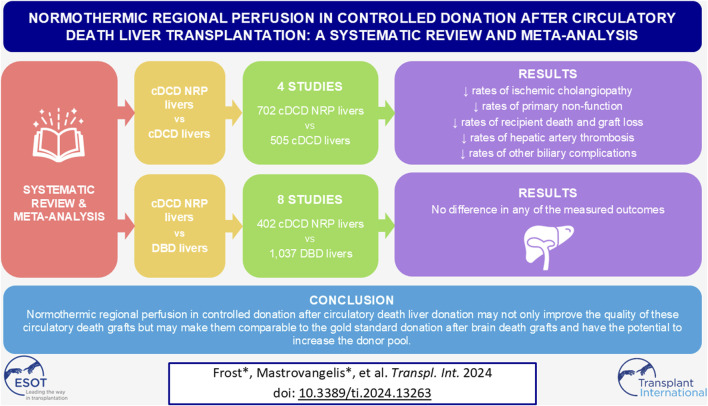

## Introduction

Due to a shortage of suitable donor livers, there is a need for expansion of the liver donor pool [1]. One proposed method of addressing this shortage has been to utilize livers from donation after circulatory death (DCD) donors. In these donors, declaration of death is made following cessation of circulation as determined by heartbeat, blood pressure, and/or electrocardiography [2]. This is followed by a super-rapid recovery procurement technique, during which the blood is flushed and the organ is cooled *in situ* prior to placement on ice. This is contrasted with donation after brain death (DBD) donors where, although the donor’s heart is still beating, brain death has been declared based on neurological criteria. DCD donors are commonly further classified as controlled (cDCD) or uncontrolled (uDCD) [3]. cDCD livers are generally considered less desirable than those recovered from DBD donors, as they are associated with higher rates of graft loss, ischemic cholangiopathy (IC), and inferior recipient survival [4, 5]. Therefore, there is significant interest in the development of novel organ procurement and preservation techniques to help improve outcomes associated with cDCD liver transplantation.

The current mainstay of organ preservation in liver transplantation is static cold storage (SCS) [6]. SCS in carefully selected DBD liver grafts have relatively low rates of known transplant complications such as early allograft dysfunction (EAD), primary non-function (PNF), and IC [6–9]. However, SCS alone in the cDCD context is associated with a higher incidence of graft complications and poorer recipient outcomes when compared with SCS in DBD livers [6]. IC is of particular concern with DCD livers (incidence of approximately 16% DCD vs. 3% DBD) [4, 10]. It has been postulated that warm ischemia (around the time of procurement) and vascular congestion contributes to microthrombus formation and subsequent biliary ischemia, leading to IC [5, 11, 12]. Compared with DBD livers, the PNF rate is greater in DCD livers (odds ratio of 3.6), as is the rate of total biliary complications (26% DCD vs. 16% DBD), and graft failure (odds ratio of 1.9) [4, 10]. These poorer outcomes contribute to higher rates of non-utilization of cDCD grafts for liver transplantation [13].

In normothermic regional perfusion (NRP) protocols, warm oxygenated perfusion with blood is restored *in situ* after declaration of circulatory death using an extracorporeal membrane oxygenation circuit. Although many technical variants exist, the circuit can be used to perfuse abdominal-only or all abdominal and thoracic organs simultaneously [13, 14]. Although the cellular mechanisms by which NRP works are not yet clear, it certainly allows for *in situ* assessment of organ function via macroscopic inspection, biopsy, and biochemical evaluation [13–16]. However, NRP does utilize more resources than super rapid recovery (SRR); including increased operating theatre time, disposables, and specifically trained perfusion staff [14].

The adoption of NRP varies significantly worldwide. It is policy to routinely use NRP in cDCD liver transplantation in Italy, France, and Norway, while also permitted for use in various other jurisdictions [13, 14, 17]. Some international centres combine NRP with additional ex-vivo machine perfusion technologies. The goal of NRP utilization is primarily to increase utilization of deceased donor organs and reduce mortality on the liver transplant waiting list. This systematic review and meta-analysis aims to compare outcomes from transplanted livers using NRP cDCD donors with non-NRP cDCD donors, as well comparing cDCD NRP outcomes with outcomes from DBD donation. We hypothesise that NRP improves the outcomes of cDCD livers and yields outcomes comparable to DBD livers.

## Materials and Methods

### Search Methods and Criteria

A literature search was conducted following the PRISMA 2020 Guidelines and was registered with PROSPERO (CRD42023432345) [18]. The databases searched included Medline, Embase and Scopus. The final search was conducted on 9th June 2023. Article screening, full text review, data extraction, and bias appraisal was conducted independently by Author 1 and Author 2. A third reviewer was used to resolve any conflicts. Covidence systematic review software (Veritas Health Innovation, Melbourne, Australia) was used for title and abstract screening as well as full text review.

The search was restricted to human studies in the English language published after 1st January 2000. The search terms focused on capturing liver transplantation and NRP. Search terms defining the comparator groups were deliberately not included to prevent over-filtering otherwise eligible studies.

Studies eligible for inclusion were randomised controlled trials and cohort studies of adult recipients of cDCD livers that had undergone NRP. Comparator groups of cDCD livers with SRR ± non-NRP machine perfusion, or DBD livers with SCS ± non-NRP machine perfusion were eligible. All indications for transplant and all MELD scores were included.

Abstracts, case reports, and systematic reviews were excluded. Studies with <5 NRP livers transplanted, NRP livers from uncontrolled donation after circulatory death (uDCD) donors, and paediatric recipients (<18 years) were excluded. Studies specifying a no-touch-time ≥5 min or containing data from jurisdictions with mandatory no-touch-times ≥5 min were also excluded [19]. The studies included in the data extraction were assessed using the Newcastle-Ottawa Scale Risk of Bias for Cohort Studies tool. Full inclusion/exclusion criteria are available in the Supplementary Material, and appraisal results are available Table 1.

**TABLE 1:** Newcastle Ottawa Scale bias appraisal.

Study	Selection				Comparability		Outcome			Quality
	1	2	3	4	1	Median follow up	1	2	3	
DeGoeij et al. [20]	**★**	**★**	**★**	**★**	**-**	23 months	**★**	**★**	**-**	Poor
Gaurav et al. [8]	**★**	**★**	**★**	**★**	**-**	38 months	**★**	**★**	**-**	Poor
Hessheimer et al. [21]	**★**	**★**	**★**	**★**	**-**	31 months	**★**	**★**	**-**	Poor
Mohkam et al., [22],	**★**	-	**★**	**★**	**★**	22 months	**★**	**★**	**-**	Good
Fernandez-delaVarga et al. [23]	**★**	**★**	**★**	**★**	**★**	23.1 months	**★**	**★**	**-**	Good
Minambres et al. [24]	**★**	**★**	**★**	**★**	**-**	6 months	**★**	-	**-**	Poor
Rodriguez et al. [25]	**★**	**★**	**★**	**★**	**-**	22.7 months	**★**	**★**	**-**	Poor
Rodriguez-Sanjuan et al. [26]	**★**	**★**	**★**	**★**	**-**	18 months	**★**	-	**-**	Poor
Ruiz et al. [27]	**★**	**★**	**★**	**★**	**★**	36 months	**★**	**★**	**-**	Good
Savier et al. [28]	**★**	**★**	**★**	**★**	**★**	34.8 months	**★**	**★**	**-**	Good
Viguera et al. [29]	**★**	**★**	**★**	**★**	**-**	>12 months	**★**	**★**	**-**	Poor

Bold values refer to scoring categories for Selection, Comparability, and Outcome domains.

**FIGURE 1:**
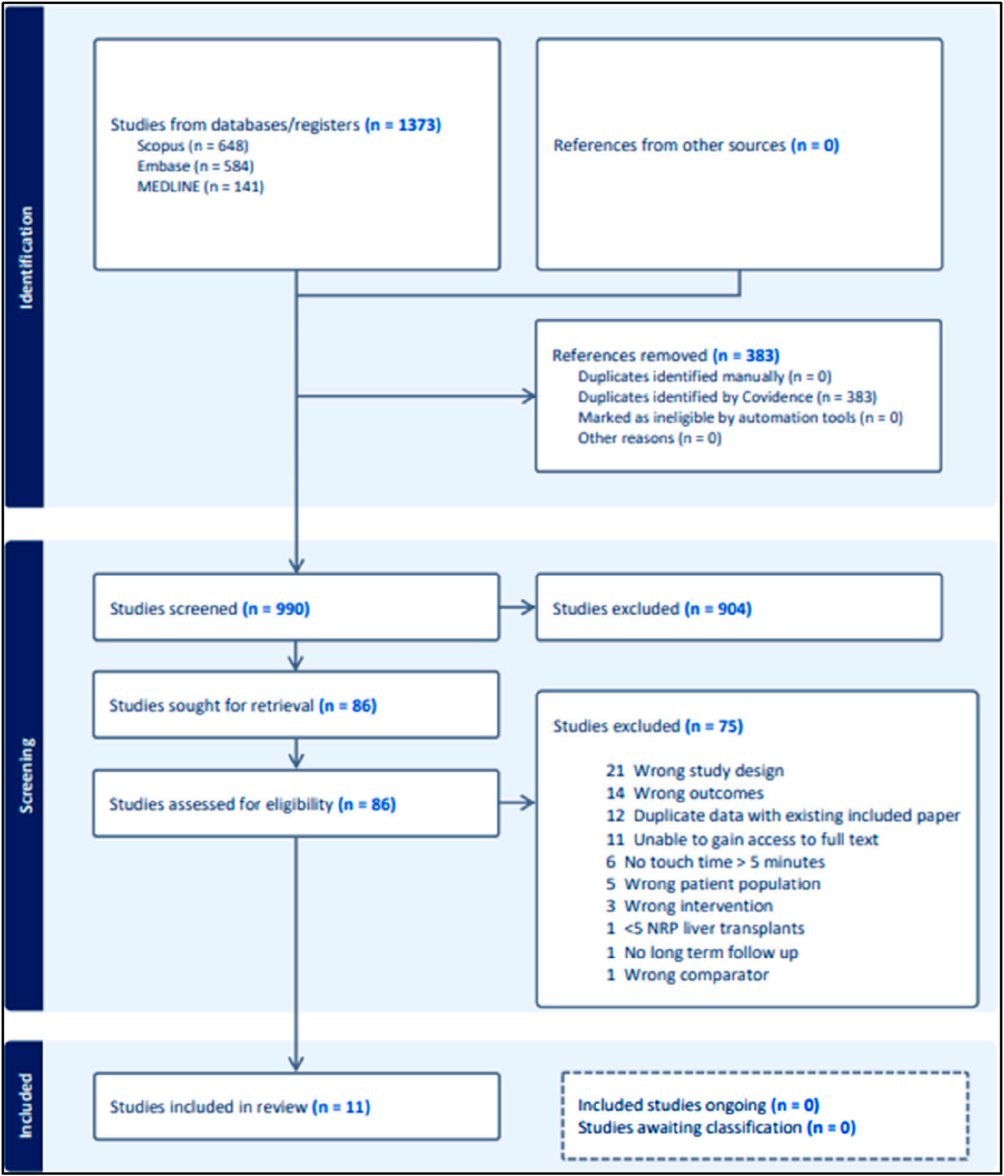
PRISMA chart.

Figure 1 is a PRISMA flow chart outlining the screening process undertaken in this review. Twelve studies were excluded from analysis due to containing duplicate data with other included studies. Preference for inclusion in these cases was given to studies published more recently and studies with higher participant numbers. Eleven studies were included in the final analysis.

### Data Extraction

Data was independently extracted by Author 1 and Author 2 into a preformed template and cross-checked. Disparities were settled with discussion and repeated review. The data extracted included number of livers transplanted, recipient death, graft loss, ischemic cholangiopathy (IC), primary non-function (PNF), hepatic artery thrombosis (HAT), early allograft dysfunction (EAD), other biliary complications, intensive care unit (ICU) length of stay, and hospital length of stay.

The following outcomes were defined for the purpose of our analysis:

• IC: non-anastomotic strictures identified through appropriate imaging with a patent hepatic artery
• PNF: graft failure leading to urgent re-transplantation or death within 1-week post-surgery
• EAD: as per Olthoff criteria [30].
• HAT: thrombosis in the hepatic artery identified through relevant imaging
• Other biliary complications: defined as anastomotic strictures and leaks, and other biliary complications identified by the study excluding IC and HAT.
• The discard rate was defined as the rate of liver grafts which were not utilized post-procurement or NRP initialisation.

### Statistical Analysis

Analysis was divided to make two separate comparisons: NRP vs. non-NRP for cDCD donation, and cDCD NRP vs. DBD donation. Further sub-group analysis was not possible due to study numbers.

Length of stay data underwent logarithmic transformation and subsequent conversion from median and interquartile range into mean and standard deviation as per Wan et al. [31] Patient death and graft loss data were analyzed by pooling hazard ratios (HR). If not reported, Kaplan-Meier plots were measured to estimate patient level survival data, which was then used to estimate hazard ratios by Cox regression. SPSS version 28.0.0.0 (IBM, United States) was used for this calculation.

Meta-analysis was performed using inverse variance random effects models. Risk ratios were calculated for dichotomous variables, mean difference was calculated for length of stay data, and hazard ratios calculated for survival data. For dichotomous variables, any study where zero events occurred in both arms was excluded. However, to ensure robustness of pooled effect, sensitivity analysis was performed by also estimating pooled effect size after continuity correction (factor of 0.5) for such studies [32]. The cut-off for statistically significant results and confidence intervals (CI) were defined as p < 0.05 and 95% respectively.

Pooled incidence of IC and PNF were estimated using the *metaprop* in Stata version 15.1 for Windows (StataCorp LLC, TX, United States) [33]. A random-effects model was used. As the incidence rates are at or close to zero for many studies, we enabled Freeman-Tukey double arsine transformation and used score confidence intervals for the individual studies. Heterogeneity was assessed using I^2^ values.

## Results

Table 1 summarises the bias appraisal of each study as per the Newcastle Ottawa Scale. Four of the studies received an overall appraisal of “good,” and seven studies received an overall appraisal of “poor.” Of these seven studies, five studies received “poor” appraisal because they did not control for confounders between the two groups and hence failed to score points in the comparability domain. Two of the included studies received a “poor” appraisal in any of the other scoring domains.

Table 2 summarises the characteristics of each study included in the NRP vs. non-NRP for cDCD donation analysis. Three of the studies utilized NRP alone, and one study utilized NRP in combination with dual hypothermic oxygenated machine perfusion (D-HOPE) for some of the transplanted livers. The comparator groups are a mix of SCS alone and in combination with machine perfusion. The number of livers transplanted in the NRP and non-NRP groups totalled 702 and 505 respectively.

**TABLE 2:** NRP vs. non-NRP for cDCD study characteristics.

Author	Year	Location	Type	Comparison	NRP livers	Non-NRP livers
Hessheimer et al. [21]	2022	Spain	Multicentre	NRP vs. SCS	545	258
Mohkam et al. [22]	2022	France/Switzerland	Multicentre	NRP vs. NMP	68	34
Gaurav et al. [8]	2022	UK	Single centre	NRP vs. SCS/NMP	69	164
De Goeij et al. [20]^a^	2022	Netherlands	Multicentre	NRP ± DHOPE vs. SCS ± DHOPE	20	49
Total					702	505

^a^ Includes 2 uDCD donations.

Table 3 summarises the characteristics of studies included in the comparison of cDCD with NRP vs. DBD donation. Two studies utilized NRP in combination with ex-vivo machine perfusion, whilst six studies utilize NRP alone. The comparator groups all utilized standard DBD techniques, except for one which utilized D-HOPE for some DBD transplants. The number of transplants in the cDCD with NRP and DBD groups totalled 402 and 1,037 respectively.

**TABLE 3:** cDCD with NRP vs. DBD study characteristics.

Author	Year	Location	Type	Comparison	cDCD livers with NRP	DBD livers
Rodriguez et al. [25]	2020	Spain	Single centre	NRP vs. DBD	39	78
Rodríguez-Sanjuán et al. [26]	2019	Spain	Single centre	NRP vs. DBD	11	51
Ruiz et al. [27]	2021	Spain	Single centre	NRP + DHOPE vs. DBD	100	200
Savier et al. [28]	2020	France	Multicentre	NRP vs. DBD	50	100
Viguera et al. [29]	2021	Spain	Multicentre	NRP vs. DBD	144	447
De Goeij et al. [20]^a^	2022	Netherlands	Multicentre	NRP ± DHOPE vs. DBD ± DHOPE	20	81
Fernandez-de la Varga et al. [23]	2022	Spain	Single centre	NRP vs. DBD	22	51
Minambres et al. [24]	2019	Spain	Multicentre	NRP Vs. DBD	16	29
Total					402	1,037

^a^ Includes 2 uDCD donations.

### cDCD NRP vs. Non-NRP

**FIGURE 2:**
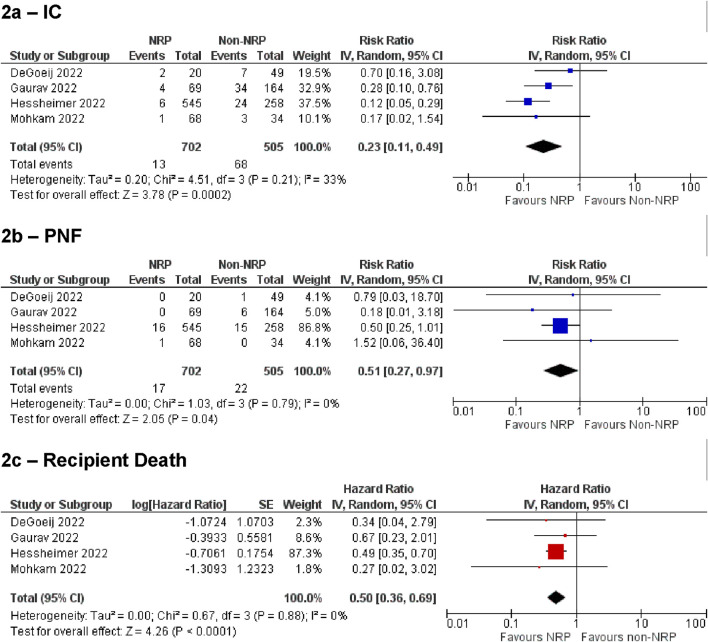
Summary of primary outcomes for NRP vs. non-NRP for cDCD. **(A)** ischemic cholangiopathy, **(B)** primary non-function, **(C)** recipient death.

Figure 2 summarises the analysis of IC, PNF, and recipient death for the NRP vs. non-NRP comparison. These demonstrated statistically significant results favouring the NRP group [IC: RR = 0.23, 95% CI (0.11, 0.49), p = 0.0002, PNF: RR = 0.51, 95% CI (0.27, 0.97), p = 0.04, recipient death: HR = 0.5, 95% CI (0.36, 0.69), p < 0.0001]. Overall incidence of IC in the NRP group was 2.6% [95% CI (0.13%–6.9%)], and 13.2% [95% CI (7.3%–21%)] in the non-NRP group. The incidence of PNF was 1.4% [95% CI (0.28%–3.0%)] in the NRP group and 3.5% [95% CI (1.7%–6.0%)] in the non-NRP group. NRP was associated with lower rates of graft loss, HAT, and other biliary complications [Graft loss: HR = 0.44, 95% CI (0.33, 0.58), p < 0.00001, HAT: RR = 0.53, 95% CI (0.31, 0.92), p = 0.02, other biliary complications: RR = 0.61, 95% CI (0.44, 0.84), p = 0.003]. There was no difference in the rate of EAD [RR = 0.78, 95% CI (0.51, 1.21), p = 0.27]. The discard rate for the NRP and non-NRP groups was 30% and 31% respectively.

### cDCD With NRP vs. DBD

**FIGURE 3:**
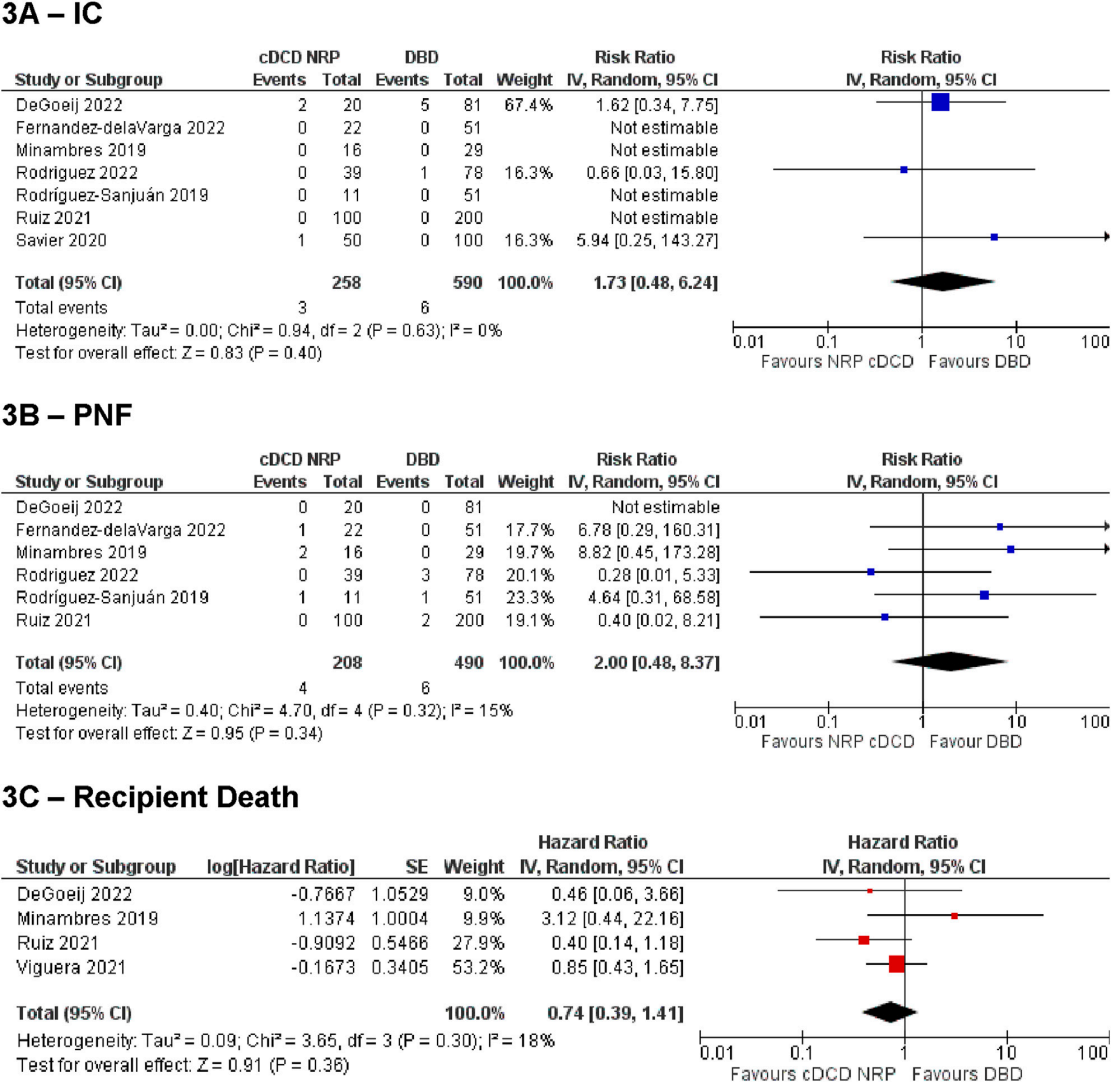
Summary of primary outcomes for cDCD with NRP vs DBD. **(A)** ischemic cholagniopathy, **(B)** primary non-function, **(C)** recipient death.

Figure 3 Summarises the analysis of IC, PNF and recipient death for the NRP vs. DBD comparison. These demonstrated no difference between the groups [IC: RR = 1.73, 95% CI (0.48, 6.24), p = 0.4, PNF: RR = 2.0, 95% CI (0.48, 8.37), p = 0.34, recipient death: HR = 0.74, 95% CI (0.39, 1.41), p = 0.36]. Sensitivity analysis by including studies with zero events on both arms (by continuity correction) confirmed these findings to be robust [IC: 1.93, 95% CI (0.66 to 5.65), p = 0.23; PNF: 2.16, 95% CI (0.62–7.52)]. The estimated overall incidence of IC was 0.13% [95% CI (0.0%–1.9%)] in the cDCD with NRP group, and 0.37% [95% CI (0.0%–2.0%)] in the DBD group. The incidence of PNF was 1.1% [95% CI (0.0%–6.2%)] in the cDCD with NRP group, and 0.69% [95% CI (0.02%–1.9%)] in the DBD group.

Statistical analysis of secondary outcomes demonstrated no difference between the two groups for any outcome [graft loss: HR = 0.75, 95% CI (0.47, 1.20), p = 0.23, HAT: RR = 0.64, 95% CI (0.24, 1.73), p = 0.38, EAD: RR = 0.94, 95% CI (0.64, 1.39), p = 0.77, other biliary complications: RR = 0.99, 95% CI (0.64, 1.53), p = 0.96, ICU stay length: MD = −0.03, 95% CI (−0.08, 0.03), p = 0.34, hospital stay length: MD = −0.07, 95% CI (−0.15, 0.02), p = 0.12].

## Discussion

The outcomes examined in this systematic review were chosen because of their clinical importance and their past association of these outcomes with DCD liver transplantation. In the comparison of the NRP and non-NRP groups for cDCD livers, NRP is unanimously associated with lower rates of IC, PNF, HAT, and other biliary complications in conjunction with lower rates of recipient death and graft loss. The discard rate in each group was comparable, suggesting that the improved outcomes seen with NRP were not due to selection bias in the discard of organs in the NRP group. The analysis of utilization is potentially confounded by the fact that the comparison is performed after a decision to proceed to donation is already made. NRP utilization is often associated with more liberal organ acceptance criteria in terms of donor age, agonal time, and graft steatosis. Grafts of poorer quality that would not commonly be utilized as part of the non-NRP denominator are compared with some livers that were considered appropriate for procurement only because NRP was available. Hence, NRP is associated with a greater overall utilization, but a similar non-utilization rate from the point of intended recovery. This parameter is not captured in the reported data, but the advantage may be inferred. Ideally, further meta-analysis on donor and recipient factors such as degree of steatosis, MELD scores, BMI, and specific NRP protocols would have been included; however, the included articles did not consistently provide this data, and the articles that did were notably heterogenous with varied graft management options in addition to NRP.

Although the heterogeneity of interventions of the included studies is recognized, we considered that this was outweighed by the benefit of such analysis in a practical sense; cDCD NRP vs. non-NRP liver transplantation studies with strictly no additional perfusion technologies are limited and are unlikely to become available in the future as it would be unethical to withhold treatment from these organs when global standards permit their use and the emerging evidence supports their effectiveness. In the clinical setting, it is also more practical to compare cDCD NRP vs. non-NRP livers that may have been treated with other perfusion technologies as this is more reflective of current practice.

In the comparison of NRP cDCD vs. DBD, NRP cDCD livers perform equally well as DBD livers, exhibiting comparable complication and survival rates. A previous systematic review by De Beule *et al.* reported an overview of NRP for liver and kidney transplantation, including cDCD transplantation [34]. That review compared outcomes of NRP against SCS for cDCD livers. Although the authors reported lower rates of IC, EAD, biliary strictures (of any type), and anastomotic biliary strictures, there was no difference in PNF, 1 year patient survival or HAT. Additionally, no comparison could be made for cDCD NRP vs. DBD livers at that time. The conclusion made by the authors was that NRP could possibly provide benefits for reducing biliary complications for cDCD donation. In our review, the rate of discard with NRP DCD was comparable to DCD liver programs around the world. Haque *et al.* describes a 30% discard rate for all DCD liver donation within the US, and Oniscu *et al.* describes a 29.6% discard rate for non-NRP DCD donation in the UK. [17, 35]. Oniscu *et al.* did however describe a lower discard rate of 18.3% for NRP DCD donation in the UK. The same study also reported a higher overall utilization rate when using NRP for liver grafts. This was attributed to two main factors; the ability for functional evaluation of organs *in situ*, and a higher acceptance rate of the initial graft offer when NRP is known to be utilized. This review has focussed on liver transplantation, however, previous analyses have shown improved post-transplant outcomes and organ utilization for other abdominal organs, such as the kidneys, when employing NRP compared to standard DCD techniques [36, 37]. All studies included in our cDCD NRP vs. non-NRP analysis reported liver transplant outcomes only, and no studies were found meeting the inclusion criteria which reported multiple organ donation outcomes. NRP circuits may be configured in a manner that allows simultaneous perfusion to multiple other abdominal and thoracic organs, allowing the potential benefits of NRP to be extended to other transplanted grafts. Further studies looking at the outcomes of multiple grafts from the same NRP donor may be beneficial.

A notable limitation of our study is that all included studies were observational, as no randomised controlled trials satisfied the inclusion criteria. The need for randomised trials to provide high quality evidence of the benefit of NRP has been previously outlined, although conducting such studies is now arguably unethical in the context of the results demonstrated above [38]. Additionally, more than half of the included studies are classified as “poor” according to the Newcastle Ottawa Scale due to the nature of the scoring system of the scale. Any paper that does not score in the comparability domain receives an automatic “poor” designation, although they may score well in all other respects. Importantly, the majority did specify that there was no statistically significant difference between the donor and recipient groups in a variety of metrics, however this demonstration is not considered sufficient to score points for comparability on the Newcastle Ottawa Scale. Another limitation is that although this review examines the use of NRP compared to non-NRP, we were unable to make any direct comparison of NRP vs. SCS, HOPE, or NMP. Hence, the outcome may be slightly confounded by livers receiving a combination of NRP, HOPE, and NMP in addition to NRP. The number of studies currently published is insufficient to facilitate direct comparisons between each technology combination. Ideally, the effect of NRP on recipient outcomes would be isolated from the effects of other ex-vivo perfusion technologies, however this is not currently possible with the available data. The control groups in each comparison (non-NRP cDCD and DBD groups) also contain liver grafts treated with HOPE or NMP in addition to standard SCS. The inclusion of these technologies in the control groups may lead to an underestimate of NRP effect. One included study contained 2 uDCD livers which could not be separated from our cDCD with NRP vs. DBD analysis. The decision was made to include this study even with the increased risk of bias, as the effect of only 2 livers in the sample size was highly unlikely to alter the results in any meaningful way and their inclusion allowed for the inclusion of 49 additional cDCD livers to increase the power of our analysis. As uDCD livers are of poorer quality, the inclusion of these livers would more likely disadvantage the NRP analysis than advantage it, making the positive effect of NRP results even more persuasive.

The most important future analysis should focus on the effect of NRP to increase utilization from the point of organ offer due to the more liberal acceptance criteria (principally on account of acceptance of more advanced donor age, longer agonal times, and higher rates of steatosis). Direct comparisons of NRP with ex-vivo machine perfusion may also be useful. It is certainly possible that some combination of NRP, HOPE, and NMP will provide the optimal combination of maximal utilization and acceptable recipient outcomes, but this will be challenging to investigate robustly on account of the possible number of combinations [13]. It should be noted that a randomised controlled trial examining NMP vs. SCS for liver transplantation demonstrated no change in biliary complication rate, graft survival, or patient survival rates whilst increasing the number of transplantable grafts by 20% [39].

In summary, this review demonstrates that the use of NRP in cDCD liver transplantation is associated with lower rates of many significant post operative complications as well as improved graft and patient survival. NRP cDCD outcomes were comparable to DBD outcomes. The use of NRP appears to also increase the utilization of cDCD livers for transplantation, although non-utilization rates of recovered DCD livers are similar between NRP and standard techniques following donation. NRP has the potential to allow for the expansion of the donor pool and improvement of outcomes so reducing the mortality for those patients needing liver transplantation.

## Data Availability

The original contributions presented in the study are included in the article/Supplementary Material, further inquiries can be directed to the corresponding author.

## Abbreviations

cDCD, controlled donation after circulatory death; D-HOPE, dual hypothermic oxygenated machine perfusion; DBD, donation after brain death; DCD, donation after circulatory death; EAD, early allograft dysfunction; HAT, hepatic artery thrombosis; IC, ischemic cholangiopathy; ICU, intensive care unit; NMP, normothermic machine perfusion; NRP, normothermic regional perfusion; PNF, primary non-function; SCS, static cold storage; SRR, super rapid recovery; uDCD, uncontrolled donation after circulatory death; WLST, withdrawal of life sustaining treatment.
