# Supplementary material for: Normothermic Regional Perfusion in Controlled Donation After Circulatory Death Liver Transplantation: A Systematic Review and Meta-Analysis
Source: Transpl Int. 2024 Aug 23;37:13263. doi: 10.3389/ti.2024.13263 (PMC11377255; doi:10.3389/ti.2024.13263)
Supplement: Supplementary file 1 [file DataSheet1.docx]

**Supplementary material**

**Inclusion/Exclusion criteria**

| **Inclusion criteria** | **Exclusion criteria** |
| --- | --- |
| Randomised controlled trials and cohort studies | Case reports, systematic reviews, letters to the editor, conference abstracts |
| NRP ± machine perfusion vs. super rapid recovery ± machine perfusion | Studies with <5 NRP livers transplanted |
| NRP ± machine perfusion vs. super rapid recovery ± machine perfusion | uDCD donors |
| NRP + ex-vivo machine perfusion/NMP | No-touch-time >5 minutes |
| cDCD donors | Non-human studies |
| All MELD scores and indications for transplant | Paediatric patients (<18 years) |

**Search Strategies**

*Medline*

Ovid MEDLINE(R) ALL <1946 to June 07, 2023>

1 Liver Transplantation/ 64101

2 ((liver* or hepat*) adj5 (transplant* or graft* or donor* or recipient*)).mp. [mp=title, book title, abstract, original title, name of substance word, subject heading word, floating sub-heading word, keyword heading word, organism supplementary concept word, protocol supplementary concept word, rare disease supplementary concept word, unique identifier, synonyms, population supplementary concept word, anatomy supplementary concept word] 102895

3 (extracorpor* adj5 oxygenat*).mp. [mp=title, book title, abstract, original title, name of substance word, subject heading word, floating sub-heading word, keyword heading word, organism supplementary concept word, protocol supplementary concept word, rare disease supplementary concept word, unique identifier, synonyms, population supplementary concept word, anatomy supplementary concept word] 22105

4 (region* and perfus*).mp. [mp=title, book title, abstract, original title, name of substance word, subject heading word, floating sub-heading word, keyword heading word, organism supplementary concept word, protocol supplementary concept word, rare disease supplementary concept word, unique identifier, synonyms, population supplementary concept word, anatomy supplementary concept word] 40760

5 NRP.mp. 1860

6 1 or 2 102895

7 3 or 4 or 5 64453

8 6 and 7 468

9 limit 8 to (english language and humans and yr="2000 -Current" and "all adult (19 plus years)") 141

*Embase*

Embase Classic <1947 to 1973>

Embase <1974 to 2023 June 07>

1 liver transplantation/ 118524

2 liver graft/ 34346

3 ((liver* or hepat*) adj5 (transplant* or graft* or donor* or recipient*)).mp. [mp=title, abstract, heading word, drug trade name, original title, device manufacturer, drug manufacturer, device trade name, keyword heading word, floating subheading word, candidate term word] 183764

4 regional perfusion/ 13255

5 (region* adj5 perfus*).mp. [mp=title, abstract, heading word, drug trade name, original title, device manufacturer, drug manufacturer, device trade name, keyword heading word, floating subheading word, candidate term word] 23116

6 (extracorpor* adj5 oxygenat*).mp. [mp=title, abstract, heading word, drug trade name, original title, device manufacturer, drug manufacturer, device trade name, keyword heading word, floating subheading word, candidate term word] 44358

7 NRP.mp. 2771

8 1 or 2 or 3 183764

9 4 or 5 or 6 or 7 69730

10 8 and 9 1171

11 limit 10 to (human and english language and yr="2000 -Current" and adult <18 to 64 years>) 584

*Scopus*

( TITLE-ABS-KEY ( liver  AND transplantation )  OR  TITLE-ABS-KEY ( ( liver*  OR  hepat* )  W/5  ( transplant*  OR  graft*  OR  donor*  OR  recipient* ) )  AND  TITLE-ABS-KEY ( extracorpor*  W/5  oxygenat* )  OR  TITLE-ABS-KEY ( region*  W/5  perfus* )  OR  TITLE-ABS-KEY ( nrp ) )  AND  PUBYEAR  >  1999  AND  ( LIMIT-TO ( DOCTYPE ,  "ar" )  OR  LIMIT-TO ( DOCTYPE ,  "cp" ) )  AND  ( LIMIT-TO ( LANGUAGE ,  "English" ) )

**Additional forest plots: NRP vs. non-NRP for cDCD donation**

| **A – Graft loss**  **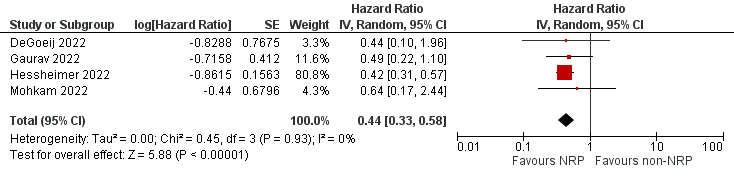**  **B – HAT**  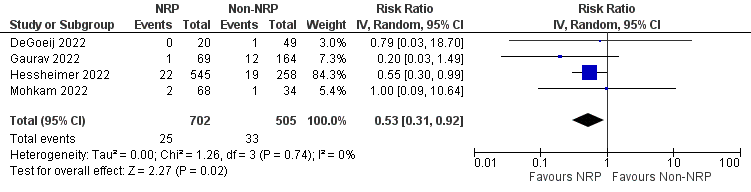  **C – Other biliary complications**  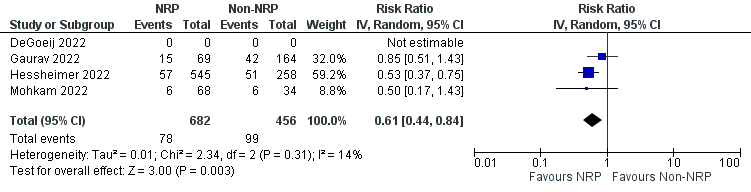  **D – EAD**  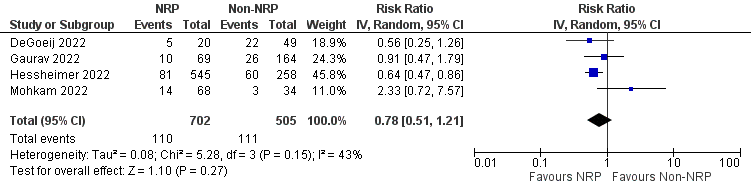 |
| --- |

**Additional forest plots: NRP cDCD donation vs. DBD donation**

| **A – Graft loss**  **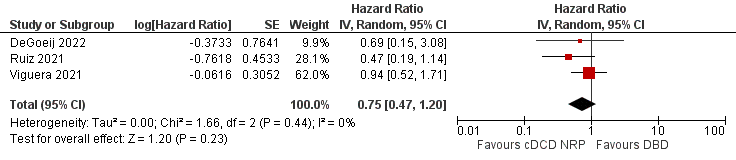**  **B – HAT**  **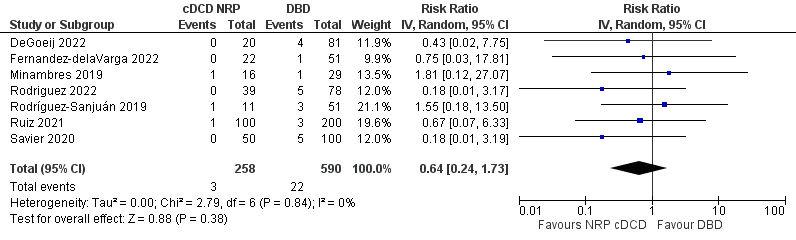**  **C – Other biliary complications**  **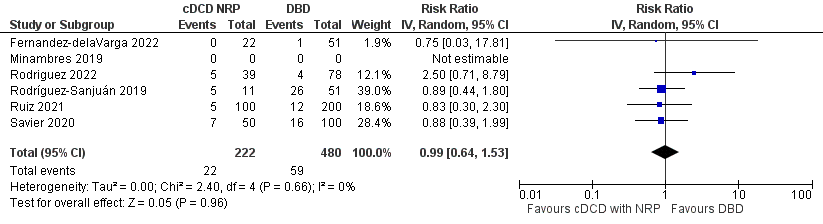**  **D – EAD**  **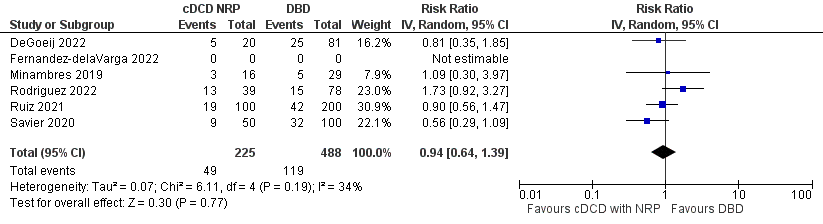**  **E – Hospital length of stay**  **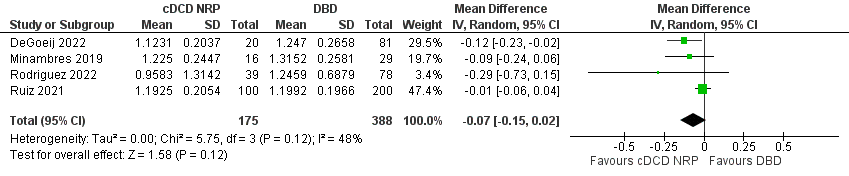**  **F – ICU length of stay**  **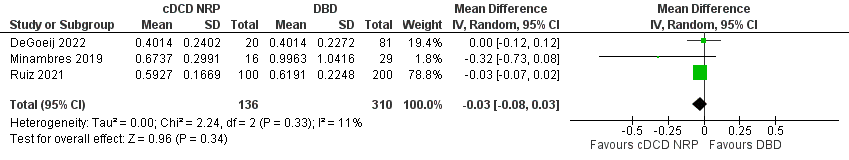** |
| --- |

**Funnel plots NRP vs. non-NRP**

| **a) Ischaemic cholangiopathy**   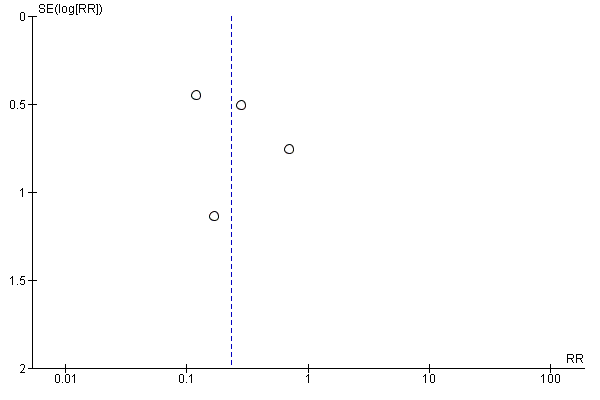  **b) Primary non-function**   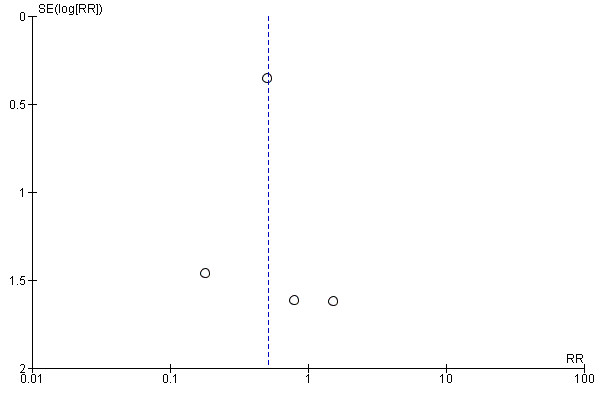  **c) Recipient Death**   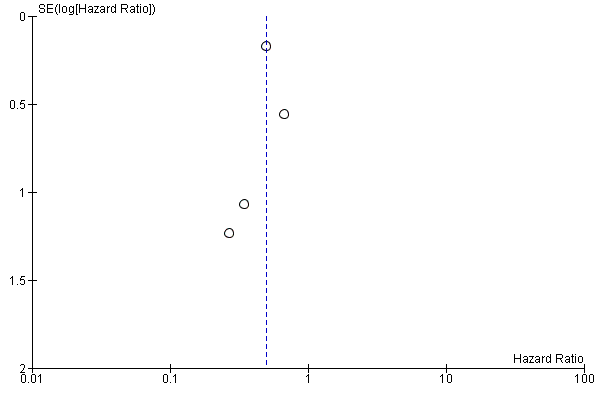  **d) Hepatic artery thrombosis**   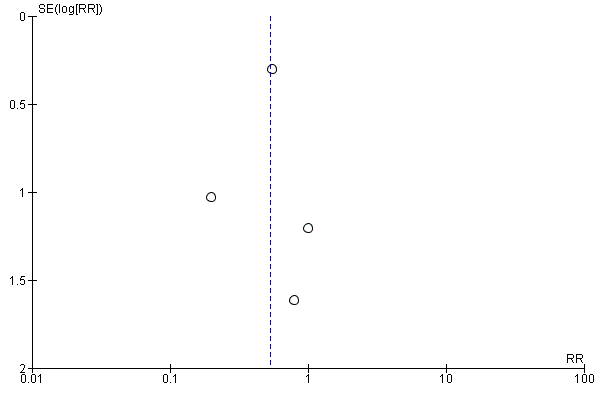 | **e) Early allograft dysfunction**   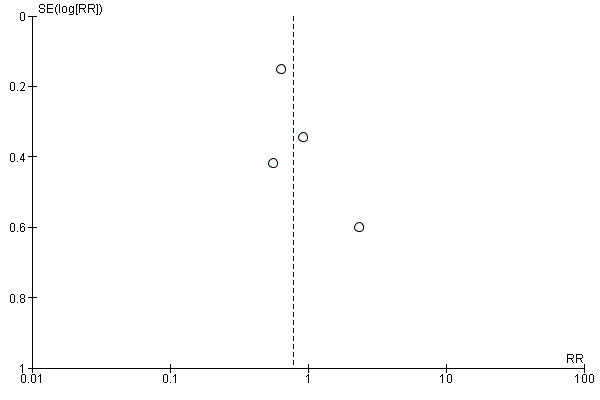  **f) Other biliary complications**   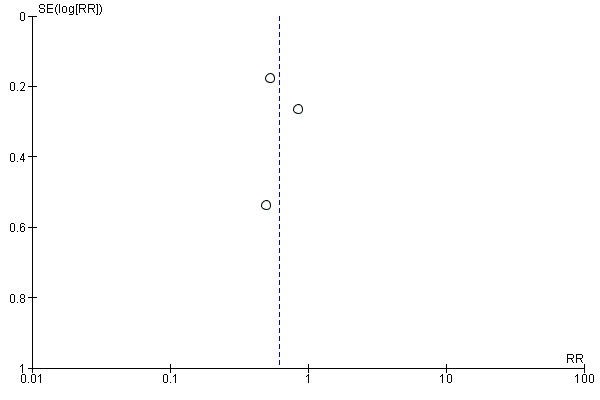  **g) Graft loss**   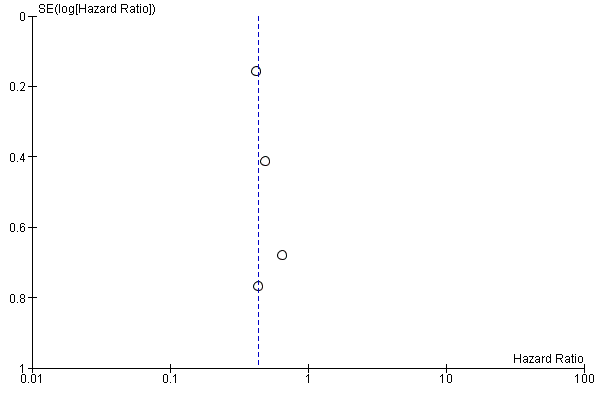 |
| --- | --- |

**Funnel plots cDCD NRP vs. DBD**

| **a) Ischaemic cholangiopathy**   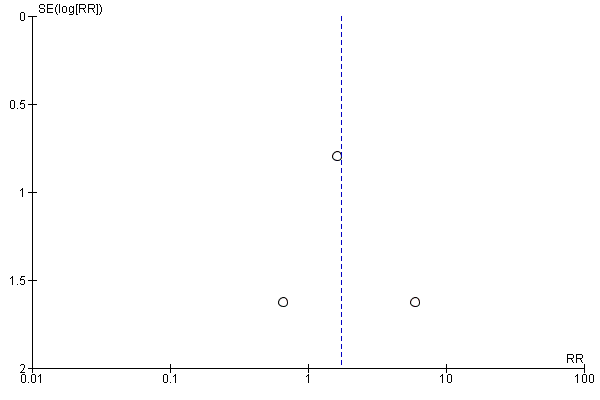  **b) Primary non-function**   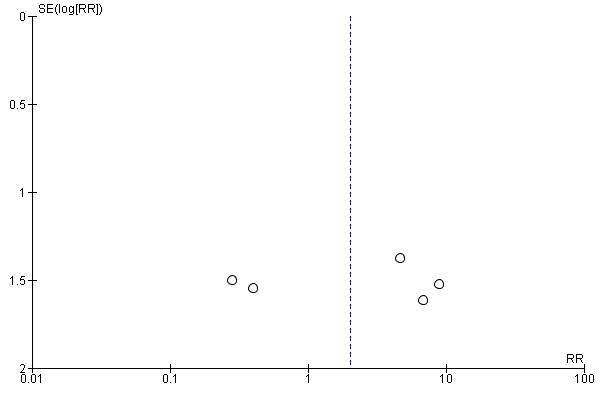  **c) Recipient Death**   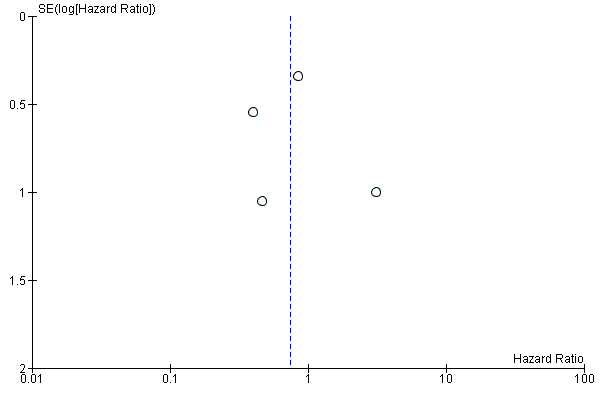  **d) Hepatic artery thrombosis**   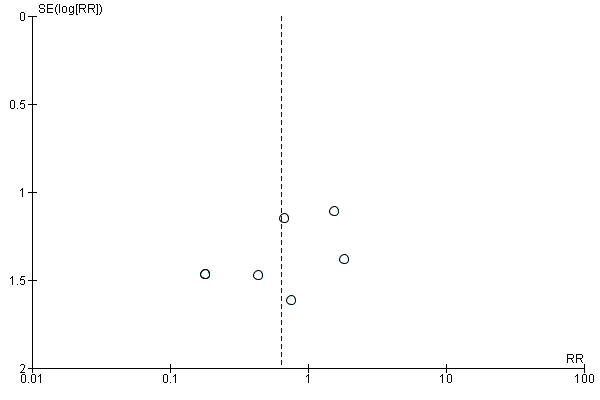  **e) Early allograft dysfunction**   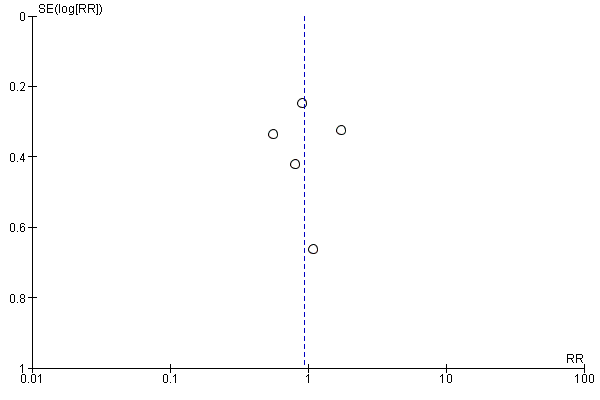 | **f) Other biliary complications**   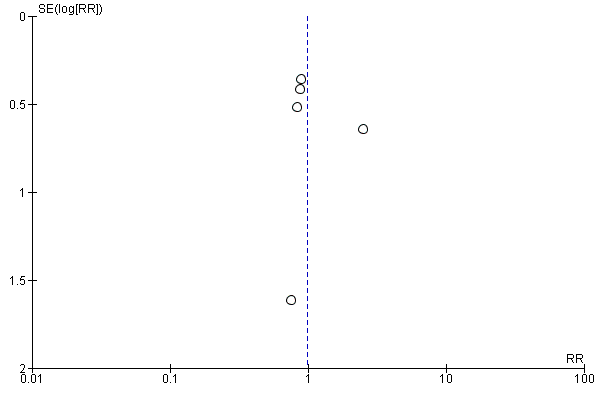  **g) Graft loss**   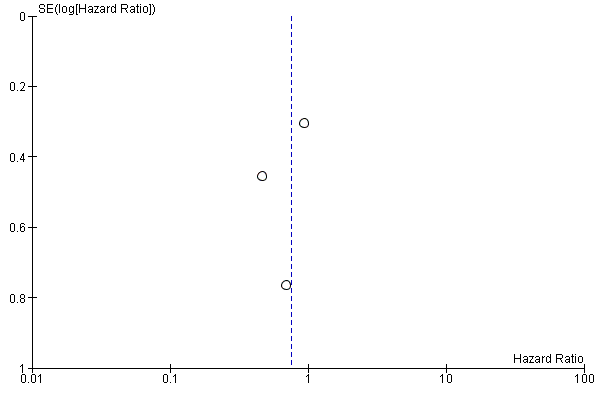  **h) ICU stay**   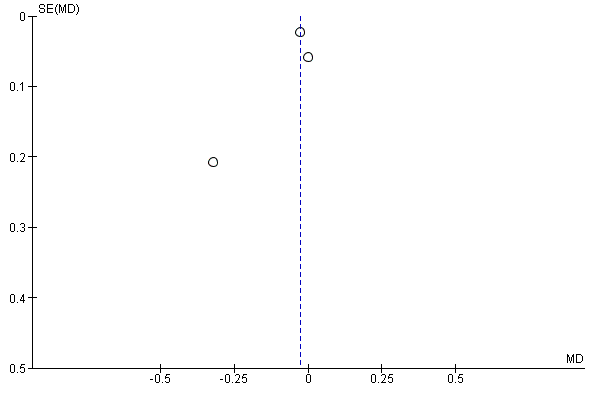  **i) Hospital stay**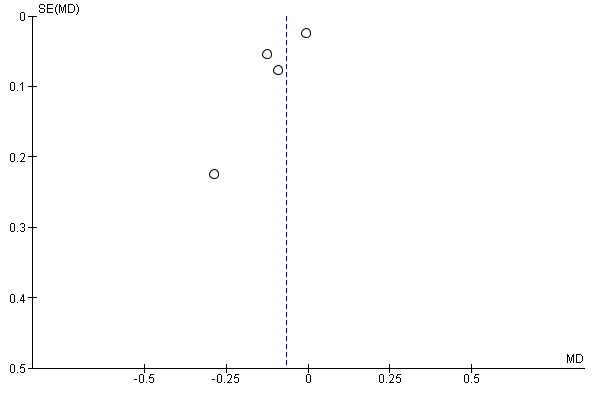 |
| --- | --- |

**MOOSE Checklist for Meta-analyses of Observational Studies**

| **Item No** | **Recommendation** | **Reported on Page No** |
| --- | --- | --- |
| Reporting of background should include | | |
| 1 | Problem definition | 5 |
| 2 | Hypothesis statement | 7 |
| 3 | Description of study outcome(s) | 8-9 |
| 4 | Type of exposure or intervention used | 6-7 |
| 5 | Type of study designs used | 7 |
| 6 | Study population | 7-8 |
| Reporting of search strategy should include | | |
| 7 | Qualifications of searchers (eg, librarians and investigators) | - |
| 8 | Search strategy, including time period included in the synthesis and key words | 7 |
| 9 | Effort to include all available studies, including contact with authors | 7 |
| 10 | Databases and registries searched | 7 |
| 11 | Search software used, name and version, including special features used (eg, explosion) | 7, 27-29 |
| 12 | Use of hand searching (eg, reference lists of obtained articles) | N/A |
| 13 | List of citations located and those excluded, including justification | 19 |
| 14 | Method of addressing articles published in languages other than English | 7 |
| 15 | Method of handling abstracts and unpublished studies | 7 |
| 16 | Description of any contact with authors | - |
| Reporting of methods should include | | |
| 17 | Description of relevance or appropriateness of studies assembled for assessing the hypothesis to be tested | 7 |
| 18 | Rationale for the selection and coding of data (eg, sound clinical principles or convenience) | 12 |
| 19 | Documentation of how data were classified and coded (eg, multiple raters, blinding and interrater reliability) | 7 |
| 20 | Assessment of confounding (eg, comparability of cases and controls in studies where appropriate) | 30 |
| 21 | Assessment of study quality, including blinding of quality assessors, stratification or regression on possible predictors of study results | 30 |
| 22 | Assessment of heterogeneity | 10, 20-21, 31-33 |
| 23 | Description of statistical methods (eg, complete description of fixed or random effects models, justification of whether the chosen models account for predictors of study results, dose-response models, or cumulative meta-analysis) in sufficient detail to be replicated | 9-10 |
| 24 | Provision of appropriate tables and graphics | 15-20, 29-35 |
| Reporting of results should include | | |
| 25 | Graphic summarizing individual study estimates and overall estimate | 19-20, 30-32 |
| 26 | Table giving descriptive information for each study included | 15-16 |
| 27 | Results of sensitivity testing (eg, subgroup analysis) | 11 |
| 28 | Indication of statistical uncertainty of findings | 10-11 |

| **Item No** | **Recommendation** | **Reported on Page No** |
| --- | --- | --- |
| Reporting of discussion should include | | |
| 29 | Quantitative assessment of bias (eg, publication bias) | 33-35 |
| 30 | Justification for exclusion (eg, exclusion of non-English language citations) | 7 |
| 31 | Assessment of quality of included studies | 29 |
| Reporting of conclusions should include | | |
| 32 | Consideration of alternative explanations for observed results | 13-14 |
| 33 | Generalization of the conclusions (ie, appropriate for the data presented and within the domain of the literature review) | 14 |
| 34 | Guidelines for future research | 14 |
| 35 | Disclosure of funding source | 2 |

*From*: Stroup DF, Berlin JA, Morton SC, et al, for the Meta-analysis Of Observational Studies in Epidemiology (MOOSE) Group. Meta-analysis of Observational Studies in Epidemiology. A Proposal for Reporting. *JAMA*. 2000;283(15):2008-2012. doi: 10.1001/jama.283.15.2008.
